# Amplify, Amplify: Shotgun Proteomics Boosts the Signal for Biomarker Discovery

**DOI:** 10.1289/ehp.117-a206

**Published:** 2009-05

**Authors:** Angela Spivey

Disease processes or exposure to environmental toxicants can produce tiny modifications (called adducts) on proteins in the blood. A clinical assay that reliably detects those modifications in plasma or serum could confirm environmental exposures or speed diagnosis of diseases such as cancer. But scientists have identified so many protein adducts that might serve as candidate biomarkers that finding the best ones to advance into further testing presents a major challenge. Advancements in an approach known as shotgun proteomics now promise to streamline the discovery process by identifying the most promising biomarkers for exploration.

Scientists are still laying the rudiments of a systematic “pipeline” for biomarker discovery, says Dan Liebler, a professor of biochemistry, pharmacology, and biomedical informatics at Vanderbilt University School of Medicine. Liebler has for almost 10 years used proteomics to study protein damage caused by oxidative stress or chemical toxicity induced by reactive endogenous chemicals. “We have knowledge of [reactions or changes] that could be advanced at some point to biomarkers, but there are so many candidate changes in tissues or living systems exposed to environmental stressors, and we don’t have efficient mechanisms for identifying the best possible markers to move forward in the pipeline,” Liebler says.

In the last 10 years proteomics technologies involving mass spectrometry have made it possible to quickly identify proteins and quantify adducts, down to the very amino acid site of modification, so scientists can more quickly screen potential biomarkers. In a typical shotgun proteomics approach, a scientist would take a biologic sample, add enzymes to digest all the proteins to peptides, fractionate the peptides, then analyze them on an ion trap mass spectrometer. The resulting spectra can be compared against peptide databases to determine which proteins are present in the sample.

Scientists have used the shotgun pro−teomics approach to discover many protein modifications of interest. But the list of biomarker candidates must be narrowed by determining which ones can be reproducibly measured in large numbers of clinical samples, such as blood samples from unexposed and exposed people. Performing such tests of candidate bio−markers currently requires development of targeted immunoassays, which is difficult and expensive because scientists have to develop an antibody for use in the assay that is specific to the protein of interest.

Improvements in mass spectrometry assays have the potential to change that. Proteomics researchers now predict that in as little as three to four years hybrid immuno–mass spectrometry assays, which combine some elements of traditional immunoassays with some elements of shotgun proteomics, will be used to evaluate candidate bio−markers in a large number of samples.

## An Assay for Organophosphate Exposure

One common proteomic approach to identifying biomarkers involves exposing an *in vitro* system to a source of damage such as a particular chemical, then using a mass spectrometer to identify the protein modifications. That is the basic approach used by Mike MacCoss, an assistant professor of genome sciences at the University of Washington, in his research with professor of medicine and genome sciences Clement Furlong. MacCoss and Furlong are searching for biomarkers of exposure to organophosphates such as tricresyl phosphate, a chemical used as a lubricant in jet engines that has been measured in aircraft cabin air. When tricresyl phosphate is inhaled, it is metabolized into toxic cyclic saligenin phosphate. A definitive measure of exposure to this chemical could help determine whether such exposure is the source of neurologic symptoms such as tremor and memory loss that have been reported among airline workers.

In an attempt to develop an assay for organophosphate exposure, MacCoss and colleagues use a combination of protein biochemistry and proteomics. “The way protein biochemistry was done previously is that you would have an activity you were interested in, or you had an antibody recognizing a given protein, and as you went about purifying your protein, you’d use the activity to trace [the protein], and then you’d go about determining what your protein sequence is,” MacCoss says. “We’re doing the same thing,” he explains. “It’s just that we’re using a mass spectrometer on the back end to speed that process up.”

Organophosphate compounds are known to inhibit a family of proteins called carboxylesterases. To find specific proteins of interest, MacCoss and colleagues first perform activity assays with serum samples *in vitro*. An organophosphate compound is added to the sample, then a traditional biochemical activity assay is used to track proteins that are modified by the compound. The scientists continue to track activity and fractionate the sample until they have purified it, meaning it contains only one protein. At this point they do not know the identity of the protein, but they do know that its activity has been modified by the addition of the organophosphate.

Next the researchers use the purified sample to perform a more targeted experiment using microcapillary liquid chromatography–tandem mass spectrometry to identify the protein of interest and to measure the modifications induced by the original organophosphate addition. To perform this experiment, they add an enzyme such as trypsin to digest the protein to peptides. “That produces a series of overlapping peptides that span the entire protein sequence,” MacCoss says.

The researchers can identify both the protein and the exact sites of modification because a modification will cause a slight shift in the peptide’s mass. Using this process, MacCoss and colleagues have identified three candidate proteins that are modified by organophosphate compounds, and they have identified the site of modification on each protein. Now MacCoss—and other scientists who have similarly found candidate biomarkers—must test them in a larger number of samples, such as tissue or serum from exposed individuals.

## Finding the Tiniest Trees in the Forest

One major challenge in testing candidate biomarkers *in vivo* is that the modified protein will be present in a typical patient sample in a very tiny concentration, even tinier than in the *in vitro* experiments. “The real challenge is being able to see the smaller, less abundant modified proteins that are going to be more informative, versus the ‘redwoods’—or abundant proteins—that will be everywhere in the forest of blood proteins,” says B. Alex Merrick, a staff scientist in the NIEHS Laboratory of Respiratory Biology. Finding biomarkers of specific environmental exposures can be especially difficult compared with identifying biomarkers of disease. “A lot of times you’re down at the noise level looking for changes,” Merrick says. “It’s more difficult to prove [modification by exposure].”

MacCoss tries to address that problem by making affinity reagents that will enrich serum samples for the protein of interest without going through the laborious purification used during the *in vitro* experiments. “The affinity reagent will preferentially bind one protein over other ones, so you effectively make it so that your protein of interest is a greater proportion of the total proteins left over in your mixture,” he says. “Then we will follow that by a mass spectrometry step, and that will allow us to make the process much faster.” MacCoss predicts he will be able to do the affinity enrichment in parallel for many samples at once, and spectrometry assay.

## Hybrid Assays

Right now, according to Liebler, mass spectrometry provides a “bridging technology” that can help scientists sort through biomarker possibilities without having to develop a high−quality, robust assay such as an immuno−assay to evaluate each potential biomarker. But evaluating biomarker candidates in a large group of patient samples still requires developing a targeted immunoassay for those specific biomarkers. “It’s very hard and expensive to make really good antibodies to do high−quality ELISAs—the standard platform for immunoassay,” Liebler says.

But Liebler predicts that mass spectrometry–based assays could soon advance to the point that they are used in place of standard immunoassays to evaluate biomarkers. “It’s likely that mass spectrometry will be able to take a place alongside immunoassays in conducting marker trials, as an analytical platform of choice,” Liebler says.

Such a hybrid immuno–mass spectrometry assay would use an antibody (the “immuno” part) to enrich the blood sample for the protein of interest, then the “mass spectrometry” part identifies whether the protein of interest has been modified and to what degree. The hybrid assay would still require development of an antibody, but the antibody would not need to perform at the level required for today’s standard immunoassays.

“With a hybrid immuno–mass spectrometry assay, the antibody . . . gives the mass spectrometry–based detection a boost by enriching the target from a very complex mixture,” Liebler says. He and others are working with such assays in the research setting, but Liebler is not aware of work with such an assay being published yet in the literature. “These assays are really not ready for prime time now,” he says. But he predicts that within the next three to four years they’ll be used in research studies to validate biomarkers in large numbers of patient samples.

MacCoss has also developed software to improve the ability of mass spectrometry to detect the lower−abundance proteins in a sample. In an article published in the 3 April 2009 issue of the *Journal of Proteome Research*, for example, MacCoss and colleagues described their use of an algorithm that programs the mass spectrometer to preferentially acquire spectra of the peptides that are in lower abundance, writing, “Our approach uses the high peak capacity of the mass analyzer to resolve and detect peptide features that would not normally be sampled in the presence of more intense interfering signals.” MacCoss says, “It’s not perfect, but it’s an improvement. It would work for a case in which a small fraction of your sample contains a modified peptide and you had enough material to run the analysis multiple times.”

Another challenge for any future application of clinical assays that identify bio−markers of exposure is that proteins—and any modifications that have happened because of exposure—might degrade in the body before a patient is tested. “As soon as a protein becomes degraded, we won’t be able to measure the modified form of that protein,” MacCoss says. “Some of the protein biomarkers that we’re following have pretty short half−lives. That’s something we’ll have to work out when we get there.” In the meantime, proteomics researchers and their use of hybrid immuno–mass spectrometry technologies for identifying protein adducts as biomarkers in exposed populations may bring us one step closer to linking environmental toxicants with illness and disease.

## Figures and Tables

**Figure:**
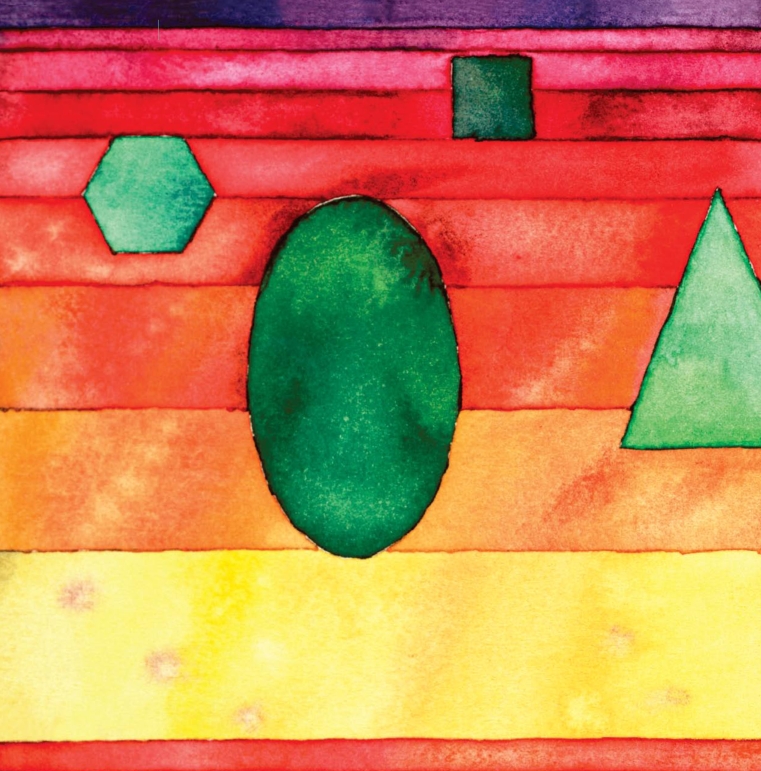


**Figure:**
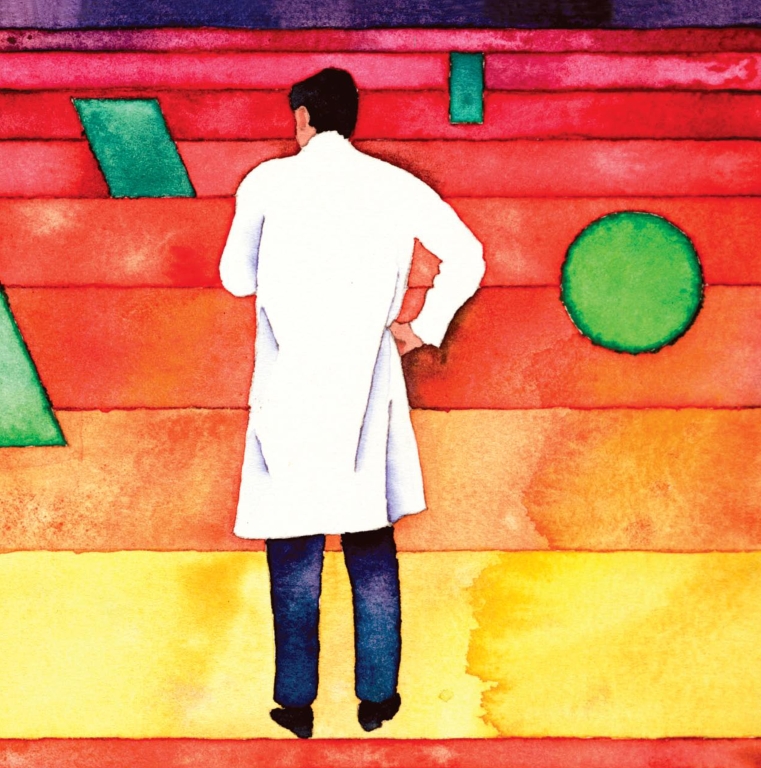


**Figure:**
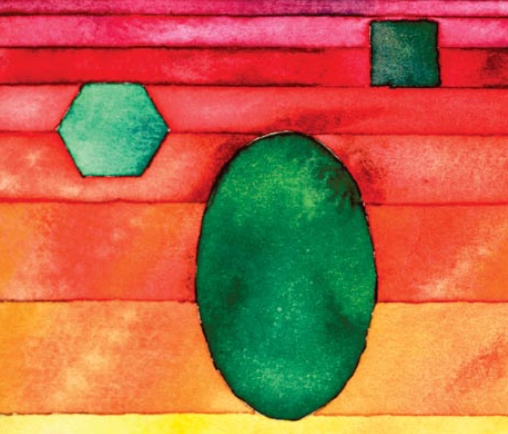
The real challenge [in testing biomarker candidates] is being able to see the smaller, less abundant modified proteins that are going to be more informative, versus the “redwoods”—or abundant proteins—that will be everywhere in the forest of blood proteins. — B. Alex Merrick NIEHS

**Figure:**
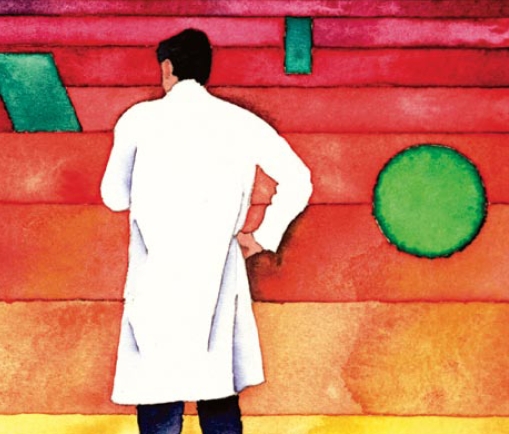
It’s likely that [mass spectrometry–based assays] will be able to take a place alongside immunoassays in conducting marker trials, as an analytical platform of choice. — Dan Liebler Vanderbilt University School of Medicine
